# Supplementary material for: Glutamate acts on acid-sensing ion channels to worsen ischaemic brain injury
Source: Nature. 2024 Jul 10;631(8022):826–34. doi: 10.1038/s41586-024-07684-7 (PMC11269185; doi:10.1038/s41586-024-07684-7)

**Supplementary Methods**

**Chemical synthesis of LK-1 and LK-2**

*Synthesis of LK-1*

*Scheme:*

*Synthesis of 4-(phosphonomethyl)piperazine-2-carboxylic acid (LK-1).*

Step 1：Synthesis of 1-benzyl 2-methyl 4-((diethoxyphosphoryl)methyl)piperazine-1,2-dicarboxylate (Compound 1). K_2_CO_3_ (1.24 g, 8.98 mmol) was added to a mixture of diethyl (iodomethyl)phosphonate (1.50 g, 5.39 mmol) and 1-benzyl 2-methyl piperazine-1,2-dicarboxylate (500 mg, 1.80 mmol) in DMF (10 ml). The resulting mixture was heated to 120 °C for 16 hours. Monitored by TLC, when reaction reached the end, the reaction mixture was cooled down to room temperature then partitioned between EA (50 ml) and H_2_O (50 ml). The organic phase was separated, washed with brine (30 ml), dried over Na_2_SO_4_, filtered and concentrated under reduced pressure to dryness. The resulting residue was purified by Prep-TLC (SiO_2_, PE: EA = 2:1) to afford 1-benzyl 2-methyl 4-((diethoxyphosphoryl)methyl)piperazine-1,2-dicarboxylate as yellow oil. Yield: 128 mg (17%). m/z: ES^+^ [M+H]^+^ = 429.2.

Step 2：Synthesis of 4-(phosphonomethyl)piperazine-2-carboxylic acid (LK-1).

1-benzyl 2-methyl 4-((diethoxyphosphoryl)methyl)piperazine-1,2-dicarboxylate (120 mg, 280.10 μmol) was dissolved in concentrated hydrochloric acid (6 N, 5 ml). The reaction mixture was heated to 100 °C for 16 hours. Monitored by TLC, when reaction reached the end, solvent was removed by rotary evaporator under reduced pressure. Resulting residue was triturated with MeCN (10 mL) at 25 °C for 15 min, after filtration, 4-(phosphonomethyl)piperazine-2-carboxylic acid was obtained as light yellow solid. Yield: 40 mg (64%).m/z: ES^+^ [M+H]^+^ = 225.00 1H NMR (400 MHz, DMSO-d6) δ 4.33-4.04 (m, 2H), 3.91-3.79 (m, 1H), 3.73-3.21 (m, 6H).

*Synthesis of LK-2*

*Scheme:*

*Synthesis of 4-(phenyl(phosphono)methyl)piperazine-2-carboxylic acid (LK-2)*

Step 1: Synthesis of 1-benzyl 2-methyl 4-((dimethoxyphosphoryl)(phenyl)methyl)piperazine-1,2-dicarboxylate. Benzyl 2-methyl piperazine-1,2-dicarboxylate (330 mg, 1.19 mmol) was dissolved in acetonitrile (15 ml), benzaldehyde (138 mg, 1.3 mmol) and dimethyl phosphite (193.3 mg, 1.54 mmol) were added. The reaction mixture was heated to 80 °C for 48 h. Monitored by TLC, when reaction reached the end, solvent was removed by rotary evaporator under reduced pressure. The resulting residue was purified by column chromatography (SiO_2_, DCM/MeOH= 20:1) to obtain 1-benzyl 2-methyl 4-((dimethoxyphosphoryl)(phenyl)methyl)piperazine-1,2-dicarboxylate. Yield: 400 mg (70%). m/z: ES^+^ [M+H]^+^ = 477.1

Step 2: Synthesis of 4-(phenyl(phosphono)methyl)piperazine-2-carboxylic acid (LK-2). 1-benzyl 2-methyl 4-((dimethoxyphosphoryl)(phenyl)methyl)piperazine-1,2-dicarboxylate (400 mg, 0.840 mmol) was dissolved in concentrated hydrochloric acid (6 N, 20 mL), then reaction mixture was heated to 90 °C for 24 hours. Monitored by TLC, when reaction reached the end, the reaction mixture was cooled down to room temperature, concentrated under reduced pressure by rotary evaporator to dryness. The resulting residue was purified by reverse-phase chromatography to obtain 4-(phenyl(phosphono)methyl)piperazine-2-carboxylic acid as a white solid with the following conditions: column, C18, 40 g, 20-35 μm, 100 Å; mobile phase, CH_3_CN:H_2_O (0.05% NH_3_H_2_O) = 5: 95;

Detector, UV 200 nm. Yield: 170 mg (68%). m/z: ES^+^ [M+H]^+^ = 301.1

^1^H NMR (400 MHz, D2O) δ 7.52-7.36 (m, 5H), 4.48-4.40 (m, 1H), 4.30-4.08 (m, 2H), 3.79-3.54 (m, 2H), 3.46-3.22 (m, 3H).

**Supplementary Discussion**

**The effect of Ca^2+^ and Zn^2+^ on ASIC1a**

Because Ca^2+^ can block ASIC1a, glutamate might indirectly attenuate the action of Ca^2+^, unmasking the inhibition of I_ASICs_. To address this, we generated the ASIC1a mutant with ablated Ca^2+^ blocking site (*h*ASIC1a^E427G/D434C^). Despite decreased amplitude and increased proton affinity of *h*ASIC1a^E427G/D434C^ mutant, glutamate potentiated its currents with comparable magnitude to those seen in wildtype *h*ASIC1a (Extended Data Fig. 1f, g). Reducing extracellular concentration from 1 mM to 0.1 mM did not affect the magnitude of glutamate-induced potentiation or left-shift in EC_50_ (Extended Data Fig. 1h). Similarly, zinc (Zn^2+^) in low nanomolar concentrations can inhibit ASICs by a different site from calcium, potentially allowing glutamate to disinhibit Zn^2+^block. However, I_ASICs_ remained potentiated by glutamate in the presence of Zn^2+^ chelator, *N*,*N*,*N′*,*N′*-tetrakis(2-pyridinylmethyl)-1,2-ethanediamine (TPEN, 10 μM) (Extended Data Fig. 1i). As a positive control, TPEN enhanced ASIC currents when 50 nM Zn^2+^ was added (Extended Data Fig. 1j-m). These results indicated that the potentiation of I_ASICs_ by glutamate was independent of the known binding sites for Ca^2+^ and Zn^2+^, and instead most likely engaged an unknown site on the ASIC1a channel. This is supported by MST assay showing a direct interaction between *h*ASIC1a^E427G/D434C^ and glutamate (Extended Data Fig. 1n), and by patch-clamp recordings of functional ASICs.

**Glutamate caused mitochondrial dysfunction via ASIC1a**

Mitochondrial dysfunction is a hallmark of excitotoxicity and an early event *en route* to neuronal death. We tested the mitochondrial membrane potential (Ψm) by an organic dye JC-1 to assess its function under different conditions (Extended Data Fig. 4d). Co-application of glutamate caused a much large drop of Ψm than pH 7.0 perfusate alone in *Asic1a*^+/+^ neurons. By contrast, *Asic1a*^-/-^ neurons did not show differences in Ψm changes following co-application of glutamate and pH 7.0 (Extended Data Fig. 4e-g).

**MD simulations on glutamate-*c*ASIC1a complexes**

We performed 50-ns long molecular dynamics (MD) simulations on glutamate-*c*ASIC1a^K379^ (wild-type) and glutamate-*c*ASIC1a^K379A^ mutant complexes to detect if any conformational changes are induced by ligand binding and to assess the structural stability of protein-ligand association (Extended Data Fig. 7d). Deviation from the starting atomic positions were evaluated using the root mean square deviation (RMSD) and root mean square fluctuation (RMSF) values extracted from MD simulation trajectory during 50 ns simulation. The K379A mutant induced more substantial structural rearrangements of the glutamate-bound ASIC1a compared to the wildtype model, as reflected by larger RMSD fluctuations in the glutamate-*c*ASIC1a^K379A^ model (Extended Data Fig. 7h, i), while the glutamate-*c*ASIC1a model maintained more similar overall protein conformations (Extended Data Fig. 7e). In addition, the mutant complex displayed strikingly larger fluctuation of RMSF and greater fluctuations in the binding pose for glutamate (Extended Data Fig. 7f, g, j). These simulations provide additional support for K379 of *c*ASIC1a (i.e., homologous site K380 of *h*ASIC1a) being important for glutamate to bind to ASIC1a channels.

**Supplementary Tables**

**Supplementary Table 1. Optical activity of compounds.**


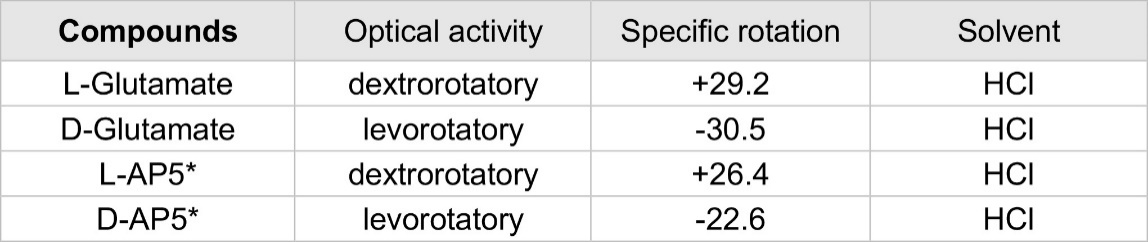


Footnote: Specific rotation of compounds (except for L-/D-AP5) was measured at 20 ℃, and the concentration was 0.1 g/ml for each compound. *Data were obtained from TOCRIS. See https://www.tocris.com.

**Supplementary Table 2. Glutamate-*c*ASIC1a binding site screening by HADDOC**


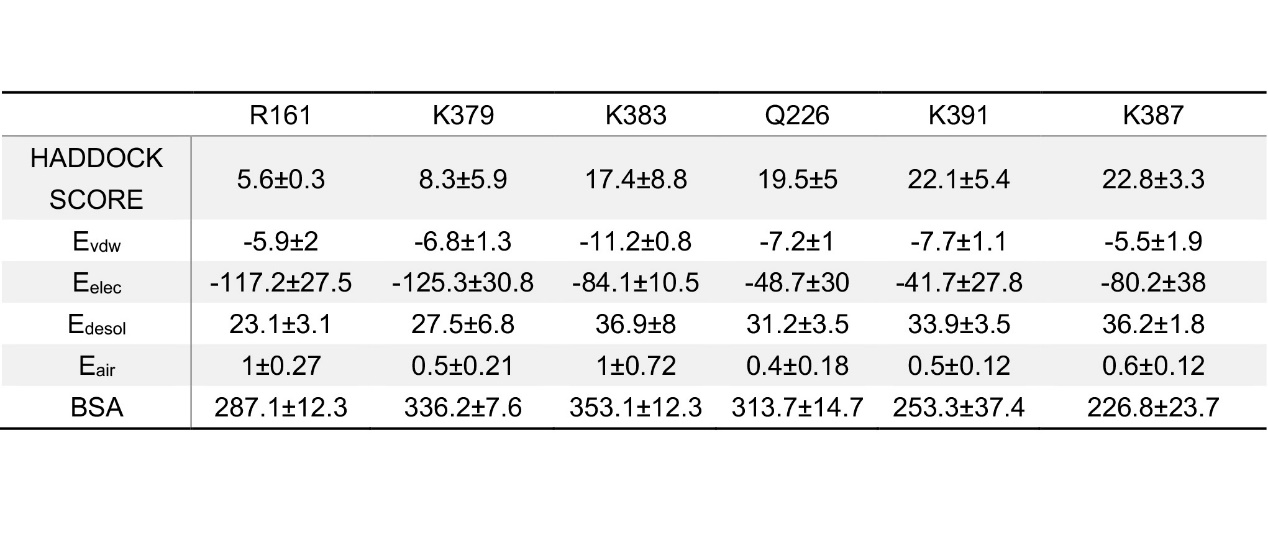


Footnote: The scoring is performed according to the weighted sum (HADDOCK score) of the following terms: Evdw: van der Waals intermolecular energy; Eelec: electrostatic intermolecular energy; Edesol: desolvation energy; Eair: ambiguous interaction restraint energy; BSA: buried surface area.

**Supplementary Table 3. Impact of K379A mutation on stability of cASIC1a structure**


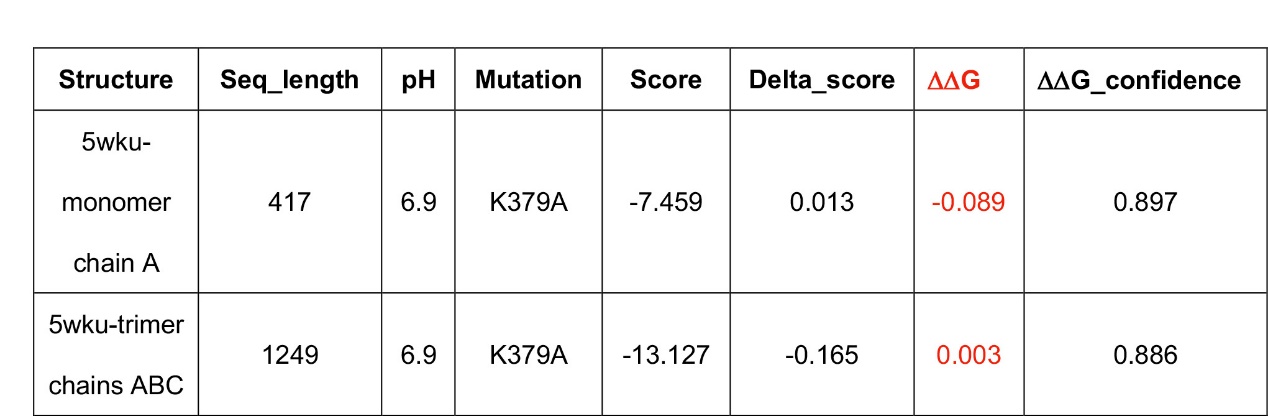


Footnote: The stability was evaluated by using MAESTRO [Laimer, J., Hofer, H., Fritz, M., Wegenkittl, S. & Lackner, P. MAESTRO--multi agent stability prediction upon point mutations. BMC Bioinformatics 16, 116, doi:10.1186/s12859-015-0548-6 (2015).], a structure-based method for predicting protein stability upon a mutation in a monomeric or multimeric form. The stability impact is summarized as a predicted free energy change (ΔΔG) with prediction confidence given as ΔΔG_confidence.

**Supplementary Table 4. CGS19755 binding pocket in *c*ASIC1a.**

**
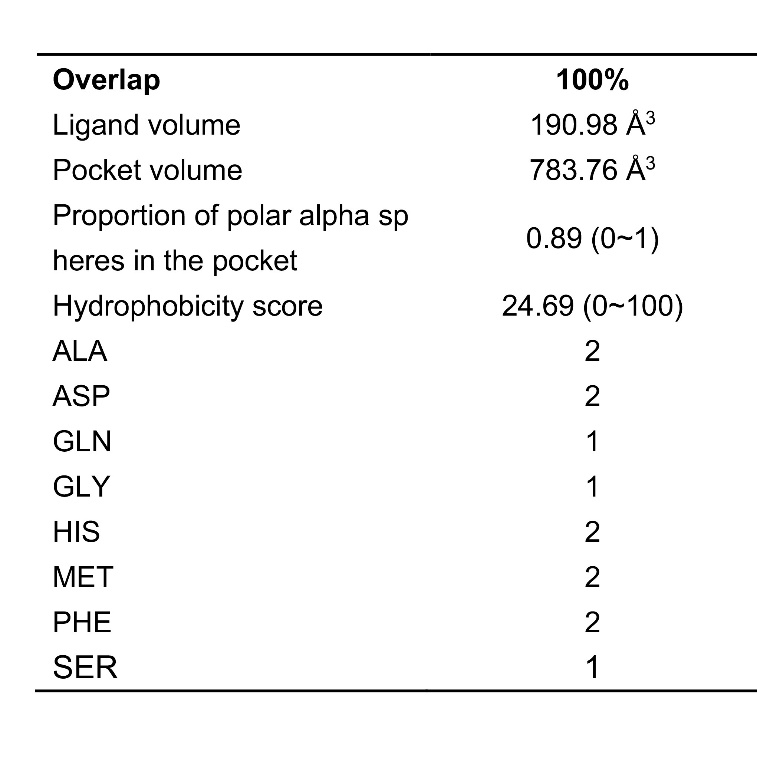
**

**Supplementary Table 5. LK-2 pharmacokinetic parameters in plasma and brain after a single intraperitoneal administration to male C57BL/6 mice.**

**
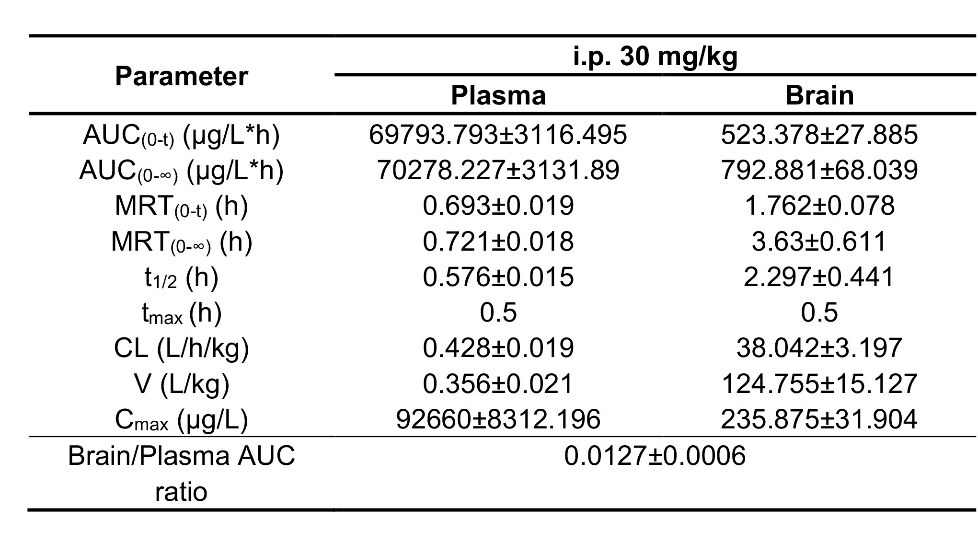
**

Footnote: AUC: area under the concentration-time curve; MRT: mean residence time; t_max_: time to maximal concentration; t_1/2_: terminal elimination half-life; CL: clearance rate; V: apparent volume of distribution; C_max_: maximal concentration.

**Supplementary Table 6. Physiological parameters of MCAO mice.**


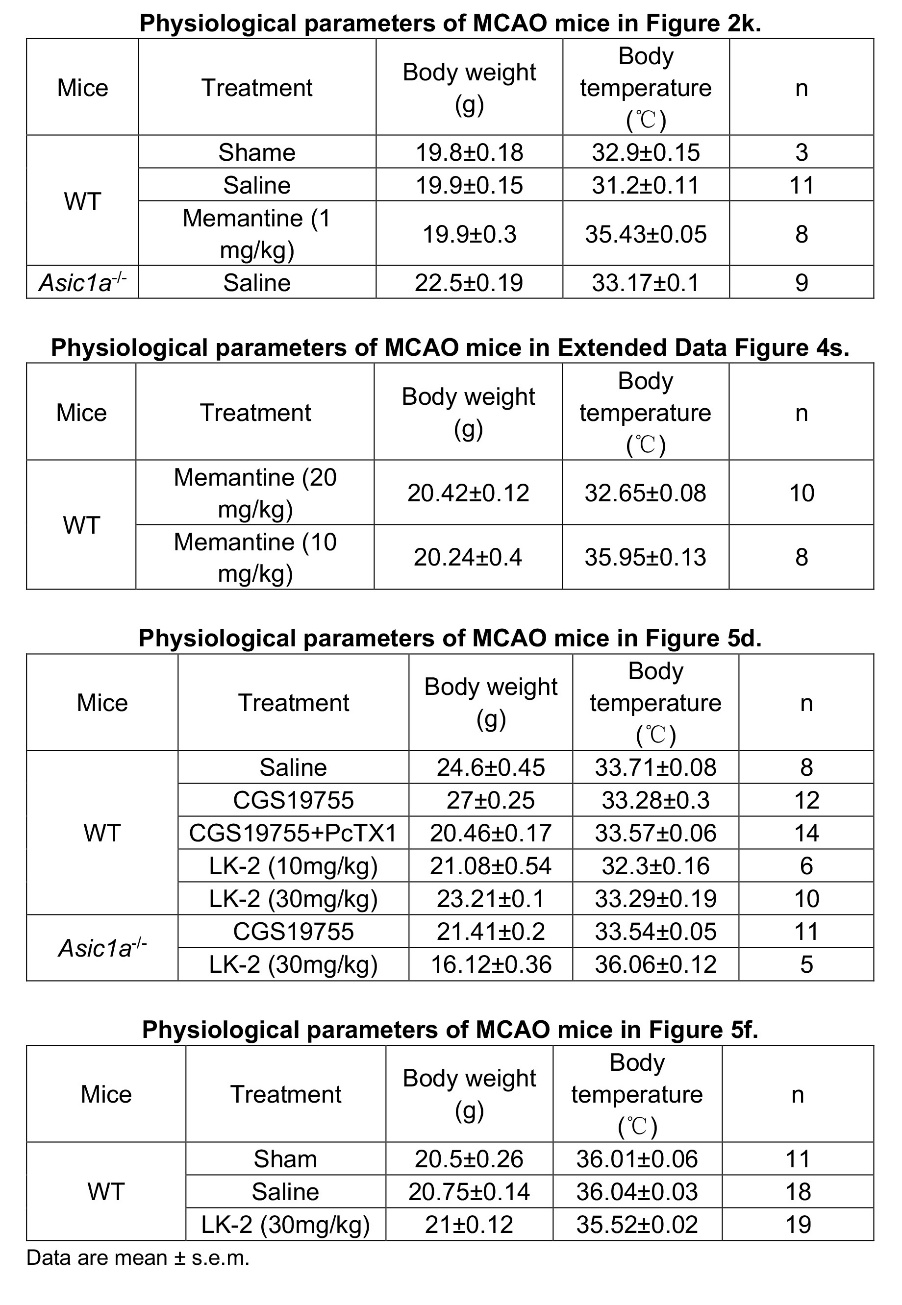

Supplement: Supplementary file 1 — Supplementary Methods, Supplementary Discussion and Supplementary Tables 1–6. Description of the synthesis and the physical characterization of LK-1 and LK-2. Detailed description of results from Extended Data Figs.1f–n, 4d–g and 7d–j. [file 41586_2024_7684_MOESM1_ESM.docx]
