# Supplementary material for: Glutamate acts on acid-sensing ion channels to worsen ischaemic brain injury
Source: Nature. 2024 Jul 10;631(8022):826–34. doi: 10.1038/s41586-024-07684-7 (PMC11269185; doi:10.1038/s41586-024-07684-7)
Supplement: Supplementary file 2 — Reporting Summary [file 41586_2024_7684_MOESM2_ESM.pdf]

nature portfolio

Corresponding author(s): Lu-Yang Wang

Last updated by author(s): Apr 27, 2024

# Reporting Summary

Nature Portfolio wishes to improve the reproducibility of the work that we publish. This form provides structure for consistency and transparency in reporting. For further information on Nature Portfolio policies, see our Editorial Policies and the Editorial Policy Checklist.

## Statistics

For all statistical analyses, confirm that the following items are present in the figure legend, table legend, main text, or Methods section.

| n/a | Confirmed | |
|---|---|---|
| ☐ | ☒ | The exact sample size (*n*) for each experimental group/condition, given as a discrete number and unit of measurement |
| ☐ | ☒ | A statement on whether measurements were taken from distinct samples or whether the same sample was measured repeatedly |
| ☐ | ☒ | The statistical test(s) used AND whether they are one- or two-sided<br>*Only common tests should be described solely by name; describe more complex techniques in the Methods section.* |
| ☒ | ☐ | A description of all covariates tested |
| ☐ | ☒ | A description of any assumptions or corrections, such as tests of normality and adjustment for multiple comparisons |
| ☐ | ☒ | A full description of the statistical parameters including central tendency (e.g. means) or other basic estimates (e.g. regression coefficient) AND variation (e.g. standard deviation) or associated estimates of uncertainty (e.g. confidence intervals) |
| ☐ | ☒ | For null hypothesis testing, the test statistic (e.g. *F*, *t*, *r*) with confidence intervals, effect sizes, degrees of freedom and *P* value noted<br>*Give P values as exact values whenever suitable.* |
| ☒ | ☐ | For Bayesian analysis, information on the choice of priors and Markov chain Monte Carlo settings |
| ☒ | ☐ | For hierarchical and complex designs, identification of the appropriate level for tests and full reporting of outcomes |
| ☒ | ☐ | Estimates of effect sizes (e.g. Cohen's *d*, Pearson's *r*), indicating how they were calculated |

*Our web collection on statistics for biologists contains articles on many of the points above.*

## Software and code

Policy information about availability of computer code

| | |
|---|---|
| Data collection | Electrophysiology was acquired using HEKA Patchmaster software (v2x90.5); Fluorescent images were acquired using Zen Black (Zeiss). Optical density was acquired using Gen5 v3.05(BioTek); Laser speckle imagings were acquired using mFLPI2Meas v2.0 software (Moor). MST traces were acquired using MO. Control software (Ver.1.6.1). |
| Data analysis | Analysis of electrophysiology data was performed using Clampfit (v10.5). Images were analyzed by Zen Blue (Zeiss) and ImageJ (v1.51). Statistical tests were performed by Prism 8. Pymol (v2.5) were used for structural model determination, refinement. Maestro (v12.5) and HADDOCK (v2.4) were used for molecular docking. ChemBioDraw Ultra (v14.0) was used for chemical structures. MOE software (v2015.10) and Maestro (v12.5) were used for compounds screening. Molecular dynamic (MD) simulation and analysis of its results were performed by Gromacs (v2020.3) and VMD (v1.9.4). Binding pocket analysis was performed by Fpocket 2.0 software. MM-PBSA calculations on MD simulation trajectories were performed with a modified gmx_mmpbsa bash script (available at Github). MST data were analyzed by MO. Affinity analysis software (Ver.2.3) of NanoTemper. Animal behavior data were collected and analyzed by Smart software (v3.0.06) of Panlab. |

For manuscripts utilizing custom algorithms or software that are central to the research but not yet described in published literature, software must be made available to editors and reviewers. We strongly encourage code deposition in a community repository (e.g. GitHub). See the Nature Portfolio guidelines for submitting code & software for further information.

# Data

Policy information about availability of data

All manuscripts must include a data availability statement. This statement should provide the following information, where applicable:

- Accession codes, unique identifiers, or web links for publicly available datasets
- A description of any restrictions on data availability
- For clinical datasets or third party data, please ensure that the statement adheres to our policy

Source data are provided with this paper.

# Field-specific reporting

Please select the one below that is the best fit for your research. If you are not sure, read the appropriate sections before making your selection.

☒ Life sciences ☐ Behavioural & social sciences ☐ Ecological, evolutionary & environmental sciences

For a reference copy of the document with all sections, see nature.com/documents/nr-reporting-summary-flat.pdf

# Life sciences study design

All studies must disclose on these points even when the disclosure is negative.

| | |
|---|---|
| Sample size | Sample sizes were empirically determined to match or exceed typical sample sizes reported in previous studies on the topic (Xiong et al, 2004, Cell; Gao et al, 2005, Neuron). |
| Data exclusions | No data was excluded from analysis. |
| Replication | Electrophysiology data for ASIC current recordings in mice was acquired from multiple neurons in at least 3 animals of each genotype. For calcium imaging and mitochondrial membrane potential test, neurons were pooled from at least 5 mice of each genotype. For cell death experiments, data were acquired from at least 3 independent cultures for calcein-PI staining and LDH release test. For MST binding assay, at least 3 independent tests were performed. For MCAO and animal behavior studies, more than 10 animals were used for each group except the sham group (only 3 mice were used) due to the '3R' principle of animal studies. All attempts at replication were successful. |
| Randomization | Experiments were not randomized. |
| Blinding | The screening of glutamate binding site on ASIC1a and electrophysiology recordings were blind to researchers until mutations of binding residues resulted in ablation of the effect that could be readily observed by electrophysiology recordings. The biological tests of synthesized compounds in MCAO model and behavior experiments were blind to the experimenter. No other blinding was performed. |

# Reporting for specific materials, systems and methods

We require information from authors about some types of materials, experimental systems and methods used in many studies. Here, indicate whether each material, system or method listed is relevant to your study. If you are not sure if a list item applies to your research, read the appropriate section before selecting a response.

## Materials & experimental systems

| n/a | Involved in the study | |
|---|---|---|
| ☒ | ☐ | Antibodies |
| ☐ | ☒ | Eukaryotic cell lines |
| ☒ | ☐ | Palaeontology and archaeology |
| ☐ | ☒ | Animals and other organisms |
| ☒ | ☐ | Human research participants |
| ☒ | ☐ | Clinical data |
| ☒ | ☐ | Dual use research of concern |

## Methods

| n/a | Involved in the study | |
|---|---|---|
| ☒ | ☐ | ChIP-seq |
| ☒ | ☐ | Flow cytometry |
| ☒ | ☐ | MRI-based neuroimaging |

# Eukaryotic cell lines

Policy information about cell lines

| | |
|---|---|
| Cell line source(s) | The CHO K1 cells and HEK293T cells were provided by Stem Cell Bank, Chinese Academy of Sciences. |
| Authentication | The cells were routinely maintained in our laboratory. They were not authenticated for these studies. |

| | |
|---|---|
| Mycoplasma contamination | The cell lines were tested negative for mycoplasma contamination. |
| Commonly misidentified lines (See ICLAC register) | No commonly misidentified cell lines were used. |

## Animals and other organisms

Policy information about studies involving animals; ARRIVE guidelines recommended for reporting animal research

| | |
|---|---|
| Laboratory animals | Homozygous Asic1a knockout mice were originally generated from Jackson Laboratory, wild-type mice of the same background (C57BL/6j) were purchased from Jiesijie company (China) and housed in our laboratory. |
| Wild animals | This study did not involve wild animals. |
| Field-collected samples | This study did not involve samples collected from the field. |
| Ethics oversight | All experimental protocols and animal use were approved by the Ethics Committee of Shanghai Sixth People's Hospital. |

Note that full information on the approval of the study protocol must also be provided in the manuscript.
